# Supplementary material for: Research on plasma arc flame length detection technology based on region of interest
Source: PLoS One. 2025 Apr 3;20(4):e0321110. doi: 10.1371/journal.pone.0321110 (PMC11967929; doi:10.1371/journal.pone.0321110)
Supplement: Attachment — Submitted filename: Response_to_Reviewers_auresp_2.docx [file pone.0321110.s004.docx]

List of Responses

Dear Editors and Reviewers,

Thank you for giving us the opportunity to submit a revised draft of the manuscript “PONE-D-24-07571R1” for publication in the PLOS ONE. We appreciate the time and effort that you and the reviewers dedicated to providing feedback on our manuscript and are grateful for the insightful comments on and valuable improvements to our paper. We have incorporated most of the suggestions made by the reviewers. Those changes are highlighted in the manuscript. Please see below, in blue, for a point-by-point response to the reviewers’ comments and concerns. All page numbers refer to the revised manuscript file with tracked changes.

**Editors**

1. The language and tutorial need to be improved. It is suggested to seek help from a native English speaker or assistance from a formal proofreading service. For instance,

a.In the abstract, the following sentence describes the main problem and is yet hard to read: “Traditional plasma rotary electrode legal powder technology of servo into the feed speed is artificially set, can not guarantee the end of the powder and plasma gun distance constant, easy to lead to the quality of the metal powder is affected and there is a large safety hazard, for these problems,”

Response to comment: In the revised manuscript, we have modified this section, please read the abstract section for details.

Abstract: With the rapid advancement of metal 3D printing technology, there is a growing demand for spherical metal powder as a primary material for 3D printing. The process technology that ensures the production of high-quality spherical metal powder has become a focal area of research for numerous enterprises and research institutions globally. In the conventional plasma rotating electrode method for powder production, the feed speed of the servo feeding mechanism is manually predetermined, leading to potential variations in the distance between the end face of the metal rod and the plasma gun that generates the plasma arc. Such inconsistency can compromise the quality of the metal powder produced and pose safety hazards if the gap between the metal rod and the plasma gun is too narrow. To address these issues, this study presents a novel plasma arc length detection system based on the concept of the region of interest. The proposed system leverages image processing technology for efficiently detecting the plasma arc length. By incorporating image detection within the region of interest alongside an arc length correction function, the system enhances real-time performance and detection precision. Additionally, real-time monitoring of the detection site is enabled through KingView. Experimental findings indicate that the image target area post plasma arc detection exhibits well-defined edges, clear brightness, and minimal noise, thereby meeting the prerequisites for subsequent image processing and monitoring tasks. The corrected plasma arc length averages around 40mm, with a detection error of less than 1mm when compared to the desired controlled plasma arc length. Moreover, the length variation remains relatively stable, thus fulfilling the measurement criteria. Over time, the detected plasma arc length exhibits negligible fluctuations, suggesting consistent proximity between the plasma gun and the end face of the metal rod during the melting process. The controller can dynamically control the feed speed of the servo feeding mechanism according to the detected plasma arc length, ensuring a constant distance between the plasma arc and the end face of the metal rod throughout the powder production process, thus aligning with practical industrial requirements.

b. The commas and periods need to be appropriately used throughout the manuscript.

Response to comment:Thank you very much for your suggestion. In the latest manuscript, we have carefully reviewed the use of commas and periods and made modifications in the latest manuscript.Please read the follwing:

Muhammad et al. [8] presented a flame identification method utilizing the GoogleNet model [9].

During this process, precise control of the plasma arc is essential as any fluctuations can jeopardize the consistency of the metal powder and potentially lead to safety hazards. To address these challenges, we have devised a metal powder device that incorporates a plasma arc flame length detection system.

The first section is the Introduction, which provides a concise overview of the content and significance of the paper.

Lens selection is contingent upon magnification and focal length requirements. The desired lens magnification should adhere to the formula: camera pixel count * vertical resolution / detection field of view = 0.12. Additionally, the focal length of the lens is determined by: working distance * (magnification / 1 + magnification) = 134 millimeters. A tabulated overview of the acquisition system components is presented in Table 1 for reference.

To carry out this procedure, a ruler is positioned adjacently to the plasma arc flame, while maintaining a constant camera angle. Subsequently, an image of the ruler is captured and subjected to the same pre-processing steps detailed previously.

The identified target area's boundary within the plasma arc flame appears well-defined with a smooth edge, exhibiting a clear and noise-free brightness level.

The identified target area's boundary within the plasma arc flame appears well-defined with a smooth edge, exhibiting a clear and noise-free brightness level. This ensures suitability for requisite image processing and monitoring tasks.

Figure 8 portrays the corrected results, showcasing the efficacy of the formula in refining the detected length.

As can be seen from Figure 7, the corrected plasma arc flame length is about 40mm.

Figure 9 illustrates the lengths of the plasma arc flames detected during three separate intervals within the same experimental setup, each spaced 20 minutes apart.

c. A few reference works have been cited using square brackets “[]” while for others, round brackets “()” have been used. Please use a consistent form according to the PLOS ONE suggested referencing style.

Response to comment:Thank you very much for your correction. In the revised manuscript, we have followed the citation style recommended by PLOS ONE and uniformly cited references using square brackets “[]”. Please read the follwing:

As the global manufacturing sector upgrades, manufacturing is growing faster than ever. Notably, 3D printing technology [1] stands as a representative of advanced manufacturing techniques that have significantly impacted the development of the manufacturing industry. In the realm of 3D printing, metal powder serves as a key material [2]. The main methods for preparing metal powders include plasma rotating electrode preparation (PREP), plasma atomization (PA), and aerosol gas atomization (GA) techniques [3]. Among these methods, the plasma rotating electrode approach is highlighted for its ability to produce spherical metal powder with minimal oxygen content and high cleanliness [4]. In the plasma rotating electrode method, the process of preparing metal powders is typically carried out in a controlled gas environment of high-purity argon to prevent oxidation and impurities. Despite its advantages in producing high-quality metal powders, challenges such as empirical setting of the metal rod feed speed and the instability of the plasma arc can impact the consistency and safety of the process. Maintaining a constant distance between the metal rod and the plasma gun to ensure optimal powder quality is crucial, as deviations in this distance can lead to safety hazards and operational issues, such as explosions or back spray incidents.

Extensive research has been dedicated to enhancing flame detection technologies. Jiang B et al. [5] proposed a method that combines local texture features and global color features of the LAB histogram to achieve swift and accurate flame detection. Similarly, another study [6] utilizes a classifier to pinpoint the flame's target area through the extraction of texture features in the flame region. With the rapid evolution of deep learning technologies, neural networks have also found application in the sphere of flame detection. Frizzi et al. [7] introduced a flame detection approach based on convolutional neural networks, which exhibits effective detection capabilities; however, the detection accuracy varies significantly when encountering flames of different colors. Muhammad et al. [8] presented a flame identification method utilizing the GoogleNet model [9]. This method incorporates transfer learning to fine-tune model parameters, striking a balance between detection efficiency and accuracy, albeit resulting in a reduction in detection accuracy. In a separate document [10], a video-based flame recognition method was proposed. By employing multi-scale fusion to extract flame features, the detection rate was enhanced, significantly boosting real-time performance. Furthermore, a study [11] introduced a flame identification algorithm based on an enhanced version of the YOLOv5 network [12]. Leveraging transfer learning [13], this method enhances the small target detection layer, augments the model's correlation identification capacity, and ultimately improves flame detection accuracy. While these methods have shown promise in detecting parameters such as flame shape, category, and area, they fall short in capturing length information of the flame.

Some researchers have explored the detection of plasma arc in various studies. A noteworthy piece of literature [14] employs spectroscopy as a technique to assess plasma arc, utilizing blackbody radiation to analyze prominent spectral lines and determine the temperature of the plasma arc. Prasad et al. [15] introduced a simulation-based approach to quantify the plasma current within the International Thermonuclear Experimental Reactor (ITER). Their method involved investigating the polarization rotation induced by Faraday effect in a rotating fiber optic sensor situated around the vacuum chamber, accounting for bending and twisting effects to assess the reflectometer's performance in gauging plasma current in the ITER facility. Another scholarly work [16] utilized a pair of synchronized high-speed cameras to capture the dynamics of both the plasma column and tracer particles. This study conducted a comprehensive 3D data analysis of the column and tracer particles, encompassing the reconstruction of the plasma column in three dimensions and the measurement of tracer particles' velocities using discrete tomography. By determining the 3D slip velocity and length of the plasma column, researchers were able to accurately estimate the radius of the conductive zone within the plasma column. While existing research has focused on measuring parameters such as plasma arc temperature, density, and conductivity, investigations specifically targeting the measurement of plasma arc length remain limited.

The demand for detecting plasma arc flame length in this study comprises several crucial components. Firstly, there is a need to capture the video signal of the plasma arc in real-time and accurately determine the precise position and length of the plasma arc flame. Subsequently, the identified video signal and length of the plasma arc flame should be promptly transmitted to the KingView software for the servo feed system to access the information instantaneously [19]. Finally, these data must be presented in the KingView interface with the flexibility to adjust detection parameters such as binary parameters, brightness, and filter width [20]. Consequently, the holistic framework of the plasma arc flame detection system is structured into three main segments: the acquisition system, detection system, and display system, illustrated in Figure 2.

The processes outlined in the detection system depicted in FIG. 3 correspond to the functionalities of the image processing and measurement software featured in FIG. 2. Subsequent to the actions of the acquisition system in FIG. 2, the system secures a color image of the plasma arc flame. As indicated in FIG. 3, the color image is typically initial transitioned into a grayscale format [21], following which the grayscale images undergo processing. Despite passing through the capture card, the images captured by industrial cameras often retain a considerable amount of noise [22]. Removing these noise elements is imperative for the detection goals. In this study, the approach involves employing median filtering [23] to eliminate noise within the image. Median filtering proves highly effective in eliminating discrete noise points within the image. For each given area, the algorithm selects a pixel point within that region. The median pixel value is then designated as the value of the central point, effectively eliminating point noise and preserving the sharpness of image edges, thereby enhancing overall image fidelity. To detect the plasma arc flame in the collected image, it is necessary to remove the background, extract the target flame area, and perform threshold segmentation on the image [24] to separate the target area from the background. Threshold segmentation involves determining a suitable threshold level to classify each image point as either background or target, hence delineating the target region. The threshold determination methodology is experimentally based, with the study ultimately selecting a threshold value of 200 after numerous trials to achieve optimal segmentation results. As the plasma arc flame tends to emit high brightness, small holes may persist post-segmentation. Addressing these holes is crucial to accurate plasma arc flame length calculations. Leveraging morphological operations [25], these holes can be effectively filtered out. Through multiple experiments, the study opts for a 5*5 square structural element for opening the image post-threshold segmentation. This method involves erosion followed by dilation, preserving the target area's shape while removing smaller holes without compromising the image condition. Complying with the plasma arc flame detection requirements necessitates extracting the edges within the target area. Consequently, conducting edge detection on the target area is pivotal. This study uses the canny algorithm [26] for edge detection, utilizing dual thresholds [27] to ascertain genuine and potential edges within the target region. Notably, the advantage of this algorithm is its resistance to noise interference, with the extracted edges demonstrating impressive continuity while avoiding false positives or edge loss.

d. Please elaborate what is “configuration king”
Response to comment:Thank you very much for your correction, "configuration king" was a translation error, the correct term should be "KingView". In the revised manuscript, We have modified "configuration king" to "KingView".

KingView is a comprehensive and user-friendly industrial automation monitoring system software that can collect real-time operational data from various industrial devices, such as temperature, pressure, flow, etc., and display and monitor them through intuitive graphical interfaces. With its comprehensive features and robust performance, KingView plays a significant role in the field of industrial automation, providing engineers with efficient and reliable monitoring and management tools. In this article, as shown in Figure S1, we designed and implemented the KingView display interface. The left side of the interface presents the original image of the plasma arc flame and its processed image, while the right side details the detection parameters and the detection length of the plasma arc flame.

Figure S1. Plasma arc flame detection and monitoring center system based on Kingview

2. While discussing figures 7 and 8, please explain how the “actual length” of the plasma arc was measured? While discussing equations 1 to 5, a manual measurement method using a ruler has been described. Clearly, this method cannot be used while the plasma arc is actually in operation.

Response to comment:As shown in Figure 1, the “actual length” of the plasma arc flame refers to the set distance “d” between the plasma gun and the metal rod material. Before starting the milling equipment, set the distance between the plasma gun and the metal rod material “d” at 40 mm. At the initial stage of the equipment startup, the plasma arc injected by the plasma gun reaches the end surface of the metal rod, and the “actual length” of the plasma arc is set at 40mm.

In Figures 7 and Figures 8, the length of the time eline is 50 seconds, which is intercepted from the starting pulverizing device. In the first 1 minute after the start of the equipment, because the temperature has not reached enough height, the end surface of the metal rod has not begun to melt, so the servo feeding mechanism will not start to move to the right, and the distance “d” between the plasma gun and the metal rod is maintained at 40mm. Therefore, the “actual length” of the plasma arc is the set distance of 40mm between the plasma gun and the metal rod material.

Figure 1. Metal pulverizing device based on detection of plasma arc flame length detection

Figure 7. Plasma arc flame length at different detection distances.

Figure 8. Corrected plasma arc flame length.

Regarding the use of the ruler, it should be noted that the use of the ruler in equations (2) and equations (3) in this paper is to determine the unit pixel length of the plasma arc in the image. The specific operation method is to place a ruler in the plane above the plasma arc flame to ensure that the distance from the camera to the ruler is equal to the distance from the camera to the plasma arc flame. By taking an image and using formula (2) and formula (3), the unit pixel length of the plasma arc flame in the image is calculated. Once the unit pixel length of the plasma arc flame is acquired, the ruler is no longer needed. The real length of the plasma arc flame can be calculated according to formula (4) and formula (5). When detecting the length of the plasma arc flame in the video shot, we do not need to use the ruler again, but can directly apply our calculation method to obtain the length of the plasma arc flame.

Here is the process of calculating the plasma arc flame length:

The calculation of length becomes feasible subsequent to the extraction of the boundary of the plasma arc flame. Image processing methodology enables the acquisition of the positional data for both the leading and trailing edges of the plasma arc flame: the left point $x_{left}\left(i_{1}\text{，}j_{2}\right)$and the right point $x_{right}\left(i_{2}\text{，}j_{2}\right)$. By virtue of this technology, the pixel length of the plasma arc flame can be accurately determined utilizing the following formula (1):

$l=j_{2}-j_{2}$ （1）

To carry out this procedure, a ruler is positioned adjacently to the plasma arc flame, while maintaining a constant camera angle. Subsequently, an image of the ruler is captured and subjected to the same pre-processing steps detailed previously. Through the application of formula (1), the pixel length of the ruler can be computed and denoted as N. The known physical length of the ruler, as measured directly with a measuring device, is represented as y. Thus, the real-world length of a single pixel within the ruler image is expressed in formula (2):

$c=\frac{y}{N}$ （2）

Given that the distance between the camera and the plasma arc flame is equivalent to the distance between the camera and the ruler, the physical length of the pixel unit on the ruler can be considered as the true length of the pixel unit on the plasma arc. Consequently, the accurate measurement of the plasma arc's length can be derived using the following formula (3):

$d=l*c$ （3）

In the actual detection process, the position of the camera may affect the accuracy of the plasma arc flame length detection. Therefore, it is necessary to correct the initially detected length. The actual length of the detected plasma arc flame after correction is shown in formula (4):

$d'=d*k$ （4）

where K is the correction factor, which can be represented by formula(5):

$k=\frac{d_{1}}{d_{2}}$ （5）

where

d1 is used to measure the distance between the plasma gun and the end face of the metal rod as measured by the measuring tool, which is also the actual distance of the plasma arc; and

d2 represents the length of the plasma arc flame detected before correction.

3. Time axis in figure 9 has the units of “mm”. Please explain

Response to comment:Thank you very much for your correction, the correct unit for the time axis in Figure 9 should be “S”. In the revised manuscript, we have modified the unit of the time axis in Figure 9 from “mm” to “S”.

Figure 9. Plasma arc flame length at different periods.

As shown in Figure 9, it can be judged whether the distance between the plasma gun and the metal rod end surface is kept constant by observing the plasma arc flame length changes detected in different time periods. We selected the plasma arc flame lengths of three different time periods in the same experiment, each 20 minutes apart. As can be seen from Figure 9, the plasma arc flame length is basically stable in different time periods of the same experiment, and is always controlled at about 40mm. This indicates that the distance between the plasma gun and the metal rod end surface did not change significantly during the course of the experiment. Although the metal rod melts continuously during the experiment, the controller can adjust the feed speed of the servo feeding mechanism in real time according to the detected length of the plasma arc flame, so as to ensure that the distance between the plasma arc flame and the metal rod end surface is constant during the pulverizing process to meet the control requirements.

According to the reviewer’s comments, we have revised the manuscript extensively (include grammatical errors and inappropriate descriptions). If there are any other modifications we could make, we would like very much to modify them and we really appreciate your help. We hope that our manuscript could be considered for publication in your journal. Thank you very much for your help.

We appreciate for Editors and Reviewers’ warm work earnestly, and hope that the correction will meet with approval. Once again, thank you very much for your comments and suggestions.
