# Supplementary material for: Research on plasma arc flame length detection technology based on region of interest
Source: PLoS One. 2025 Apr 3;20(4):e0321110. doi: 10.1371/journal.pone.0321110 (PMC11967929; doi:10.1371/journal.pone.0321110)
Supplement: Attachment — Submitted filename: Response to Reviewers.docx [file pone.0321110.s003.docx]

List of Responses

Dear Editors and Reviewers,

Thank you for giving us the opportunity to submit a revised draft of the manuscript “PONE-D-24-07571” for publication in the PLOS ONE. We appreciate the time and effort that you and the reviewers dedicated to providing feedback on our manuscript and are grateful for the insightful comments on and valuable improvements to our paper. We have incorporated most of the suggestions made by the reviewers. Those changes are highlighted in the manuscript. Please see below, in blue, for a point-by-point response to the reviewers’ comments and concerns. All page numbers refer to the revised manuscript file with tracked changes.

**Reviewer #1**

1. The novelty of this study is not clear. A flowchart summarizing what was done should be added to the article.

Response to comment: In the revised manuscript, We have added a flowchart to represent what we have done, please read the introduction section for details.

The novelty of this research: 1. We designed a metal powder device based on the detection of plasma arc length, changing the original control of the rod feed to control the feed of the plasma gun. By detecting the plasma arc length, we can control the feed speed of the servo feeding mechanism, keeping the distance between the metal rod end and the plasma gun constant. This is beneficial for improving the quality of the prepared metal powder and avoids the occurrence of safety issues. 2. We proposed a plasma arc length detection technology based on the region of interest, applying image processing technology to the detection of plasma arc length. We designed a plasma arc detection algorithm and a correction algorithm based on the region of interest, achieving real-time detection of the plasma arc length. This detection method does not affect the plasma arc itself and does not require placing detection devices into the plasma arc, which is of great significance for improving the safety and reliability of plasma detection.

Figure 1. Metal pulverizing device based on detection of plasma arc flame length detection

Figure 1 shows the reason why we do the plasma arc flame length detection and what we do. In the original metal pulverizing process, plasma gun injection plasma arc flame burning metal rod, metal bar end surface under the action of high-temperature plasma arc melting and the action of centrifugal force droplets, then condensation for metal powder, with the metal rod melting, servo mechanism drive metal bar transverse feed, due to the plasma arc is very unstable, without this pulverizing process cannot guarantee the metal bar end surface and plasma arc plasma gun distance constant, will affect the quality of the metal powder, and also can cause some safety accidents. Therefore, we designed the metal powder device based on the plasma arc flame length detection, the original control rod feed design to control the plasma gun feed, by detecting the plasma arc flame length to control the servo input speed, makes the metal rod end surface and plasma gun distance between constant, is beneficial to improve the quality of the metal prepared powder and avoid the occurrence of safety problems. As a special flame, a plasma arc has the characteristics of high temperature and high-intensity light, and it is difficult to detect its length by ordinary methods, to realize the measurement of plasma arc length, This study was presented based on the interest of Plasma arc flame length detection technology, will be the image processing technology Application in plasma arc flame length detection, design based on the region of interest of plasma arc flame detection algorithm and correction algorithm, realize the real-time detection of plasma arc flame length, this detection method will not affect the plasma arc itself, also need not probe into plasma arc flame, to improve the safety and reliability of the plasma detection is of great significance.

2. Authors have not provided comparison between their proposed methodology and the previous ones in the introduction. According to the current introduction, there is no contribution in the current study.

Response to comment: In the revised manuscript, We have added some references regarding plasma detection. Please read the introduction section for details.

As shown in Figure S2, after locating the position of the plasma arc flame in the image, this study designs a plasma arc flame length detection system, which can accurately and real-time measure the length information of the plasma arc flame.The system is mainly used for real-time monitoring of the plasma arc flame on-site, displaying the image of the plasma arc flame and the length of the plasma arc flame.

Figure S2. Plasma arc flame length detection system

As shown in Figures S3, Since the servo feeding mechanism is controlled by KingView software, after the detection system detects the length of the plasma arc flame, it needs to transmit the detected data and some parameter contents to KingView for display. As shown in Figures S3, we designed and implemented the KingView display interface. The left side of the interface displays the original image and the processed image of the plasma arc flame, while the right side shows the detection parameters and the length of the plasma arc flame. The identification area and position area and coordinates are settings for the region of interest. In the experiment, the position is set according to the area of the plasma arc flame in the image. The length and width of the region of interest cannot be set too large or too small; too large will affect the real-time detection, and too small will fail to detect the complete plasma arc flame.

Figure S3. Plasma arc flame detection and monitoring center system based on Kingview

3. Some figures have poor quality. The quality of the figures should be improved.

Response to comment: We have changed figure 2 and figure 3.If there are any other modifications we could make, we would like very much to modify them and we really appreciate your help.

Figure 2. Overall Architecture Design of Plasma Arc Flame Detection System.

Figure 3. Detection system process

4. Reference numbers should be increased.

Response to comment:We have added some references.

14.	Department Of Physics, York Plasma Institute University, York Plasma Institute University Department Of Physics, York Plasma Institute University Department Of Physics, York Plasma Institute University Department Of Physics, and York Plasma Institute University Department Of Physics. "Plasma Temperature Measurements Using Black-Body Radiation From Spectral Lines Emitted by a Capillary Discharge." Journal of Quantitative Spectroscopy and Radiative Transfer 220 (2018): 1-04.

15.	Prasad, Dandu, Gusarov Andrei, Moreau Philippe, Leysen Willem, Kim SungMoon, Mégret Patrice, and Wuilpart Marc. "Plasma Current Measurement in Iter with a Polarization-Otdr: Impact of Fiber Bending and Twisting On the Measurement Accuracy." APPLIED OPTICS 61, no. 9 (2022): 2406-16.

16.	Zhu, Jiajian, Jinlong Gao, Andreas Ehn, Marcus Alden, Zhongshan Li, Dmitry Moseev, Yukihiro Kusano, Mirko Salewski, Andreas Alpers, Peter Gritzmann, and Martin Schwenk. "Measurements of 3D Slip Velocities and Plasma Column Lengths of a Gliding Arc Discharge." APPLIED PHYSICS LETTERS 106, no. 4 (2015): 44101.

**Reviewer #2**

1. The authors worked on interesting research with a design of region of interest (ROI) based on image detection as well as correction function of the system. Authors further made effort to enhance the real-time performance and accuracy of detection.
   However, should try to rewrite the result section of the abstract to reflect more results that complements the technical achievements of the research beyond excerpt error margin that was stated in the abstract section.

Response to comment: Thank you very much for your compliment. In the revised manuscript, We have already rewritten the abstract section, adding a description of the results, please read the abstract for details.

Here is our rewritten Abstract: With the rapid development of metal 3D printing technology, the demand for spherical metal powder for 3D printing materials is increasing day by day, and the technology of high-quality spherical metal powder has become the key research content of many enterprises and research institutions in various countries. Traditional plasma rotary electrode legal powder technology of servo into the feed speed is artificially set, can not guarantee the end of the powder and plasma gun distance constant, easy to lead to the quality of the metal powder is affected and there is a large safety hazard, for these problems, this paper designed based on the area of interest, the image processing technology applied to the plasma arc flame length detection, through the introduction of image detection of the area of interest and arc flame length correction function, improve the real-time detection and detection accuracy, Finally, the real-time monitoring of the detection site is realized through the configuration king. The experimental results show that the edge of the image target area is smooth, has clear brightness, and no noise, which can meet the requirements of subsequent image processing and monitoring requirements. After correction, the plasma arc flame length is about 40 mm. Compared with the plasma arc flame length to be controlled, the detection error is less than 1 mm, and the length change is relatively stable to meet the measurement requirements. As changes over time, the length of the plasma arc flame changes little, and the distance between the plasma gun and the metal rod end surface with the passage of time and metal rod melting did not change significantly, the controller can according to the detection of the plasma arc flame length control the servo feed speed, makes the plasma arc flame in the process of pulverizing distance constant, meet the actual industrial production needs.

2. Keywords should be revised and sentenced should be removed and authors should only formulate keywords and not key sentences.
   The authors approach in the determination of the focal length and selection of lens based on detection requirements of the plasma arc flame is highly commendable.

Response to comment: In the revised manuscript, We have modified the keywords.

Keywords: Plasma detection, Length detection, Region of interest

3. Authors may revisit the detection system vis-à-vis the overall architectural design. Examining the components of the detection system flow which include noise filtering, threshold segmentation etc. It can be observed that these components do not accurately fit into the section indicated in the overall architecture rather the data acquisition section hence the need for more clarification in this regard so as not to confuse the readers.

Response to comment: In the revised manuscript, We have clarified the parts indicated by these components: The contents described in the detection system process in FIG. 3 are the contents of the image processing and measurement software in FIG. 2. After the action of the acquisition system in FIG. 2, the color image of the plasma arc flame is acquired. As shown in FIG. 3, the color image is usually converted into a grayscale image first, Then the grayscale images are processed.

4. Formulas may be referred to as equations and should be mentioned before the place of formulas for proper flow of reading and citing.

Response to comment：In the revised manuscript, We have referenced the formula before its appearance.

5. Since the authors' emphasis is on the region of interest, it will be of great value addition to this research to indicate dimensions and technical information of the specific area of interest in the diagrams of figures 4 and 5 in addition to the threshold. Showing the precise location and dimension of interest will improve the detection mechanism precisely. This will complement the authors information on distance analysis from the camera to the plasma arc. Showcasing these distances from the previous illustrations for the impact on detection accuracy, before Figure 6 is very important.

Response to comment:In the revised manuscript,We have explained the size information of the region of interest：Generally, we set the area of interest for the image of the size of 500 * 500 pixels。

6. Overall a good concept but the results still require a significant level of improvement to indicate how accurate and comparison with previous work done in this area is completely missing from this work.

Response to comment:We have placed some results in the supplementary materials; please refer to the supplementary materials for more information on our experimental results.

As shown in Figure S2, after locating the position of the plasma arc flame in the image, this study designs a plasma arc flame length detection system, which can accurately and real-time measure the length information of the plasma arc flame.The system is mainly used for real-time monitoring of the plasma arc flame on-site, displaying the image of the plasma arc flame and the length of the plasma arc flame.

Figure S2. Plasma arc flame length detection system

As shown in Figures S3, Since the servo feeding mechanism is controlled by KingView software, after the detection system detects the length of the plasma arc flame, it needs to transmit the detected data and some parameter contents to KingView for display. As shown in Figures S3, we designed and implemented the KingView display interface. The left side of the interface displays the original image and the processed image of the plasma arc flame, while the right side shows the detection parameters and the length of the plasma arc flame. The identification area and position area and coordinates are settings for the region of interest. In the experiment, the position is set according to the area of the plasma arc flame in the image. The length and width of the region of interest cannot be set too large or too small; too large will affect the real-time detection, and too small will fail to detect the complete plasma arc flame.

Figure S3. Plasma arc flame detection and monitoring center system based on Kingview

7. Grammar should be checked for proper construction as well

Response to comment:In the revised manuscript,we have corrected some grammatical issues.

**Reviewer #3**

1. Please correct grammar and English.

Response to comment:In the revised manuscript, we have corrected some grammatical issues.

2. I.e. in the Abstract part, there are some phrases that are not clear: the accuracy is better than the existing system detection I think not than the actual length of the plasma arc, because you mentioned 1mm, this should be the accuracy for the length but your idea is not well expressed.

Response to comment:We have rephrased these unclear sentences in the summary:After correction, the plasma arc flame length is about 40 mm. Compared with the plasma arc flame length to be controlled, the detection error is less than 1 mm, and the length change is relatively stable to meet the measurement requirements.

3. Before figure 7 you mention that the accuracy depends of the subjective consciousness. This is very interesting and should me more details about it. How did you figure it?

Response to comment:Due to the angle of the camera capturing the plasma arc flame being influenced by subjective human factors, the factors affecting the detection accuracy are significantly influenced by subjective consciousness. Therefore, we designed a correction algorithm to adjust the detection results.

4. To have a better impact, your manuscript should present also a comparison with existing method and how you can state your novel method accuracy. The manuscript must be improved.

Response to comment:In the revised manuscript,We have made improvements to the manuscript and added some references regarding plasma detection. Please read the introduction section for details.

The detection of plasma arcs has also been studied by some scholars.document(14)Using spectroscopy to measure the plasma arc, in which blackbody radiation can be applied to extremely strong lines and can be used to measure the temperature of the plasma arc. Prasad class(15)A simulation-based method was developed to measure plasma current in the International Thermonuclear Experimental Reactor (ITER) by studying the Faraday effect-induced polarization rotation in a spinning fiber placed around the vacuum vessel, accounting for bending and twisting effects, to assess the performance of the reflector for measuring plasma current under ITER.document(16)The dynamics of the plasma column and tracer particles were recorded using two synchronous high-speed cameras, providing 3D data analysis of the plasma column and tracer particles, including 3D reconstruction and 3D particle tracking speed measurement based on discrete tomography method, and the radius of the plasma column can be accurately estimated by determining the 3D slip velocity and 3D length of the plasma column. At present, there are measurements of plasma arc temperature, density, and other parameters, as well as conductive plasma, for the measurement of plasma arc length.

Figure 1. Metal pulverizing device based on detection of plasma arc flame length detection

Figure 1 shows the reason why we do the plasma arc flame length detection and what we do. In the original metal pulverizing process, plasma gun injection plasma arc flame burning metal rod, metal bar end surface under the action of high-temperature plasma arc melting and the action of centrifugal force droplets, then condensation for metal powder, with the metal rod melting, servo mechanism drive metal bar transverse feed, due to the plasma arc is very unstable, without this pulverizing process cannot guarantee the metal bar end surface and plasma arc plasma gun distance constant, will affect the quality of the metal powder, and also can cause some safety accidents. Therefore, we designed the metal powder device based on the plasma arc flame length detection, the original control rod feed design to control the plasma gun feed, by detecting the plasma arc flame length to control the servo input speed, makes the metal rod end surface and plasma gun distance between constant, is beneficial to improve the quality of the metal prepared powder and avoid the occurrence of safety problems. As a special flame, a plasma arc has the characteristics of high temperature and high-intensity light, and it is difficult to detect its length by ordinary methods, to realize the measurement of plasma arc length, This study was presented based on the interest of Plasma arc flame length detection technology, will be the image processing technology Application in plasma arc flame length detection, design based on the region of interest of plasma arc flame detection algorithm and correction algorithm, realize the real-time detection of plasma arc flame length, this detection method will not affect the plasma arc itself, also need not probe into plasma arc flame, to improve the safety and reliability of the plasma detection is of great significance.

**Reviewer #4**

1. At the end of the "Introduction" section, the organization and structure of the article should be written.

Response to comment: In the revised manuscript, we have enhanced the organization and structure of the article, as well as the information.

This article is divided into four parts, which are shown as follows:

The first part is the introduction, which gives an overview of the content and significance of this paper, analyzes the status quo related to the flame detection technology and plasma detection technology, and briefly describes the reasons and work of the plasma arc flame length of our designed detection system.

The second part is the design of the plasma arc flame length detection system. The overall detection architecture is designed according to the detection requirements of the plasma arc flame, and the scheme of the acquisition system is determined. Then a plasma arc flame length detection system based on the region of interest is designed to realize the real-time detection of plasma arc flame length.

The third part is the analysis of the experimental results, which analyzes the quality and accuracy of the plasma arc flame length. By analyzing the absence of the plasma arc flame length in practice, the effectiveness of the plasma arc flame length detection system in actual industrial production is verified.

The fourth part is divided into a conclusion, which summarizes our work and plans the direction of our future research work.

2. The abstract of the paper should be improved by adding your findings.

Response to comment: We have added explanations to the results to improve the abstract: After correction, the plasma arc flame length is about 40 mm. Compared with the plasma arc flame length to be controlled, the detection error is less than 1 mm, and the length change is relatively stable to meet the measurement requirements. As changes over time, the length of the plasma arc flame changes little, and the distance between the plasma gun and the metal rod end surface with the passage of time and metal rod melting did not change significantly, the controller can according to the detection of the plasma arc flame length control the servo feed speed, makes the plasma arc flame in the process of pulverizing distance constant, meet the actual industrial production needs.

3. The literature survey is very weak ; the critical survey must be done thoroughly by the authors to improve the paper and also add new subsection under the introduction section.

Response to comment: We have added some relevant references to improve the paper and added a new section under the Introduction.

The detection of plasma arcs has also been studied by some scholars.document(14)Using spectroscopy to measure the plasma arc, in which blackbody radiation can be applied to extremely strong lines and can be used to measure the temperature of the plasma arc. Prasad class(15)A simulation-based method was developed to measure plasma current in the International Thermonuclear Experimental Reactor (ITER) by studying the Faraday effect-induced polarization rotation in a spinning fiber placed around the vacuum vessel, accounting for bending and twisting effects, to assess the performance of the reflector for measuring plasma current under ITER.document(16)The dynamics of the plasma column and tracer particles were recorded using two synchronous high-speed cameras, providing 3D data analysis of the plasma column and tracer particles, including 3D reconstruction and 3D particle tracking speed measurement based on discrete tomography method, and the radius of the plasma column can be accurately estimated by determining the 3D slip velocity and 3D length of the plasma column. At present, there are measurements of plasma arc temperature, density, and other parameters, as well as conductive plasma, for the measurement of plasma arc length.

4. The research gap should be written clearly at the end of the introduction section from the literature survey.

Response to comment: We added some references in the introduction to describe the research gap. At present, there are measurements of plasma arc temperature, density, and other parameters, as well as conductive plasma, for the measurement of plasma arc length.

5. The conclusion section and/or experimental results section must be improved by integrating more generated results or rather stress more description on the results is necessary.

Response to comment: We have placed some results in the supplementary materials; please refer to the supplementary materials for more information on our experimental results.

As shown in Figure S2, after locating the position of the plasma arc flame in the image, this study designs a plasma arc flame length detection system, which can accurately and real-time measure the length information of the plasma arc flame.The system is mainly used for real-time monitoring of the plasma arc flame on-site, displaying the image of the plasma arc flame and the length of the plasma arc flame.

Figure S2. Plasma arc flame length detection system

As shown in Figures S3, Since the servo feeding mechanism is controlled by KingView software, after the detection system detects the length of the plasma arc flame, it needs to transmit the detected data and some parameter contents to KingView for display. As shown in Figures S3, we designed and implemented the KingView display interface. The left side of the interface displays the original image and the processed image of the plasma arc flame, while the right side shows the detection parameters and the length of the plasma arc flame. The identification area and position area and coordinates are settings for the region of interest. In the experiment, the position is set according to the area of the plasma arc flame in the image. The length and width of the region of interest cannot be set too large or too small; too large will affect the real-time detection, and too small will fail to detect the complete plasma arc flame.

Figure S3. Plasma arc flame detection and monitoring center system based on Kingview

6. There are few editorial and grammatical errors in some parts of the manuscript which can be identified easily by a careful reading. The authors can take help of native English speaker for corrections.

Response to comment: In the revised manuscript, we have corrected some grammatical issues.

According to the reviewer’s comments, we have revised the manuscript extensively (include grammatical errors and inappropriate descriptions). If there are any other modifications we could make, we would like very much to modify them and we really appreciate your help. We hope that our manuscript could be considered for publication in your journal. Thank you very much for your help.

We appreciate for Editors and Reviewers’ warm work earnestly, and hope that the correction will meet with approval. Once again, thank you very much for your comments and suggestions.
